# Corticospinal Tract Impairment of Patients With Parkinson’s Disease: Triple Stimulation Technique Findings

**DOI:** 10.3389/fnagi.2020.588085

**Published:** 2020-11-04

**Authors:** Dan Xu, Qingyun Ding, Han Wang

**Affiliations:** Department of Neurology, Peking Union Medical College Hospital, Chinese Academy of Medical Sciences, Beijing, China

**Keywords:** Parkinson’s disease, multiple system atrophy, triple stimulation technique, corticospinal tract, pyramidal sign

## Abstract

**Background:**

Corticospinal tract impairment is no longer an absolute exclusion in the updated Movement Disorder Society Parkinson’s disease criteria. Triple stimulation technique (TST) is an accurate method to quantitatively assess the integrity and impairment of corticospinal pathway in a variety of neurological diseases. This study aims to evaluate the corticospinal tract impairment in Parkinson’s disease (PD) patients using TST.

**Methods:**

Ten PD patients, 19 multiple-system atrophy parkinsonian variant (MSA-P) patients, and 12 healthy controls (HC) were sequentially recruited in this study. Information of age, disease duration, pyramidal signs, and Hoehn and Yahr (H&Y) stage was obtained from all patients. The TST was assessed at right abductor digiti minimi for HCs and both sides for patients. The Chi-square test was used for categorical variables, and variance analysis was performed for continuous variables in comparing the difference among PD, MSA-P, and HC, plus the *post hoc* tests for pairwise comparisons.

**Results:**

All subjects were age and gender matched. There was no significant difference in disease duration (*p* = 0.855), H-Y stage (*p* = 0.067), and the percentage of pyramidal signs present (*p* = 0.581) between MSA-P and PD patients. The mean TST ratio was 55.5 ± 32.2%, 81.7 ± 19.8%, and 96.8 ± 3.0% for PD, MSA-P, and HCs, correspondingly. PD patients had a significant lower TST amplitude ratio than MSA-P and HCs. The TST ratio of MSA-P was lower than HCs, but there was no significant difference (*p* = 0.160). Additionally, it was significantly higher in percentage of abnormal TST ratio between PD patients and MSA-P (*p* = 0.010).

**Conclusion:**

Corticospinal tract impairment is not a rare manifestation in PD and can be quantitatively evaluated with TST. The result needs to be verified in amplified sample.

## Introduction

Parkinson’s disease (PD) is the second most common neurodegenerative disease worldwide with rising incidence and prevalence alongside a changing population demographic (Pringsheim et al., 2014). The complicated motor and non-motor symptoms suggest extensive involvement of the central, peripheral, and enteric nervous systems (Braak and Del Tredici, 2017). PD is more like a syndrome than an entity. With the continuous development in our understanding of PD, the diagnostic criteria have been updated (Braak and Del Tredici, 2017). In 2015, the Movement Disorder Society (MDS) task force introduced the clinical diagnostic criteria for PD (Postuma et al., 2015). Several of the items differed from the United Kingdom (UK) Brain Bank criteria, which previously had been used widely. For instance, pyramidal tract impairment is recognized as one of the “red flags” for MDS-PD diagnosis, which is in contrast to the United Kingdom Brain Bank criteria that consider it an absolute exclusion (Hughes et al., 1992). This can cause confusion since multiple-system atrophy parkinsonian variant (MSA-P), a rare neurodegenerative disorder that often needs to be differentiated from PD, usually presents with pyramidal tract impairment (Gilman et al., 2008). Thus, it is worthwhile to investigate corticospinal tract impairment in both diseases.

There is some neuroimaging evidence that supports corticospinal tract impairment in PD. Over the past several years, important neuroimaging findings have revealed that PD is not confined to the nigrostriatal dopaminergic pathway and also involves the cortico-basal ganglia-thalamo-cortical neural network (Burciu and Vaillancourt, 2018). Taylor et al. (2018) observed significant increases in white matter fractional anisotropy (FA) in a widespread anatomical network that included the corticospinal tract and subcortical white matter in a 1-year longitudinal study that used diffusion tensor imaging (DTI). A meta-analysis of DTI studies in PD demonstrated that DTI was sensitive for identifying differences in the corticospinal tract between patients with PD and healthy controls (HCs) (Atkinson-Clement et al., 2017). The increased FA in the motor tract of PD suggested compensatory neuroplasticity or selective neurodegeneration (Mole et al., 2016). Evidence to support this has been reported in a transcranial magnetic stimulation (TMS) study. As a conventional electrophysiological tool, TMS was developed by Barker et al. in 1985 (Barker et al., 1985) and was used to non-invasively detect corticospinal or corticobulbar pathways (Groppa et al., 2012). Central motor conduction time (CMCT) of TMS represents the maximum conduction velocity of corticospinal axons (Cantello et al., 2002). A significant reduction in CMCT in patients with PD compared with healthy controls was first reported by Kandler et al. (1990). Other alterations of motor cortical function, such as decreased relaxed threshold and central silence period duration, have also been detected using TMS in patients with PD (Cantello et al., 2002). These neuroimaging and electrophysiological findings indicate structural and functional changes of the corticospinal tract in PD patients.

Compared with TMS, triple-stimulation technique (TST) is a more accurate method to quantitatively assess the integrity and impairment of the corticospinal pathway in a variety of diseases, such as multiple sclerosis (Magistris et al., 1999), amyotrophic lateral sclerosis (Rösler et al., 2000; Grapperon et al., 2014), and stroke (Tan et al., 2013). It has been demonstrated to be 2.75 times more sensitive than conventional TMS techniques for revealing corticospinal conduction blocks caused by severe demyelination or neurodegenerative processes (Magistris et al., 1999). The TST amplitude ratio reflects the proportion of the activated spinal motor neurons; ≥90% is considered normal, according to a previous study (Eusebio et al., 2007). In movement disorders, decreased TST ratios have been found in spinocerebellar ataxia type 6, while conventional TMS parameters were shown to be similar in controls (Sakuma et al., 2005). There have been a few studies using TST in PD patients, but none have reported abnormal TST results (Eusebio et al., 2007; Jang et al., 2014). However, given the evidence from the neuroimaging and neurophysiology studies, normal TST results in PD are surprising (Eusebio et al., 2007). A possible reason for these results is that both of the studies were performed before the MDS-PD criteria were being published (Postuma et al., 2015). Parkinsonian patients who had pyramidal signs had to be absolutely excluded from the diagnosis of PD, in accordance with the United Kingdom Brain Bank criteria (Hughes et al., 1992). To the best of our knowledge, no study since 2015 has assessed corticospinal tract involvement in PD using TST.

In this pilot study, we performed TST to quantitatively assess the involvement of the cortico-spinal tract in patients with clinically diagnosed PD, according to MDS-PD and MSA-P criteria.

## Materials and Methods

### Subjects

We prospectively recruited 19 parkinsonian variant patients of probable MSA-P who satisfied the consensus criteria (Gilman et al., 2008), 10 clinically diagnosed late-onset PD patients who fulfilled the 2015 MDS-PD criteria (Postuma et al., 2015), and 12 age-matched HCs from the neurology department of Peking Union Medical College Hospital. We obtained demographic and clinical information, including age, symptom duration, pyramidal signs, and Hoehn and Yahr (H&Y) stage. Signs of pyramidal signs were defined as a positive Babinski or Chaddock sign that was documented in the patients’ medical records. Otherwise, none of the patients had remarkable medical history that could explain the pyramidal signs. We acquired brain and spinal magnetic resonance images (MRIs). Participants who had MRI abnormalities that could affect the corticospinal pathway, such as lacunar infarctions, white matter lesions, or other lesions, were excluded from the study. All HCs had no remarkable medical history or signs on physical examination. All participants gave written informed consent.

### Triple-Stimulation Technique

Electromyography of the bilateral abductor digiti-minimi (ADM) was recorded in MSA-P and PD patients from surface electrodes using a Viking IV electromyography machine (Nicolet Biomedical, Madison, WI, United States). In HCs, electromyography of the left ADM was recorded. Bandpass filters were set at 20–2000 Hz. We performed TST using the MagPro Compact stimulator (Dantec Company, Copenhagen, Denmark) and a standard figure-of-eight TMS coil. The coil was held tangentially to the scalp, at a 45° angle from the posterior–anterior axis, with the handle pointing posterior–laterally. The motor hotspot for ADM was identified by applying single-pulse stimuli over the corresponding scalp area to evoke the largest motor-evoked potential (MEP).

**FIGURE 1:**
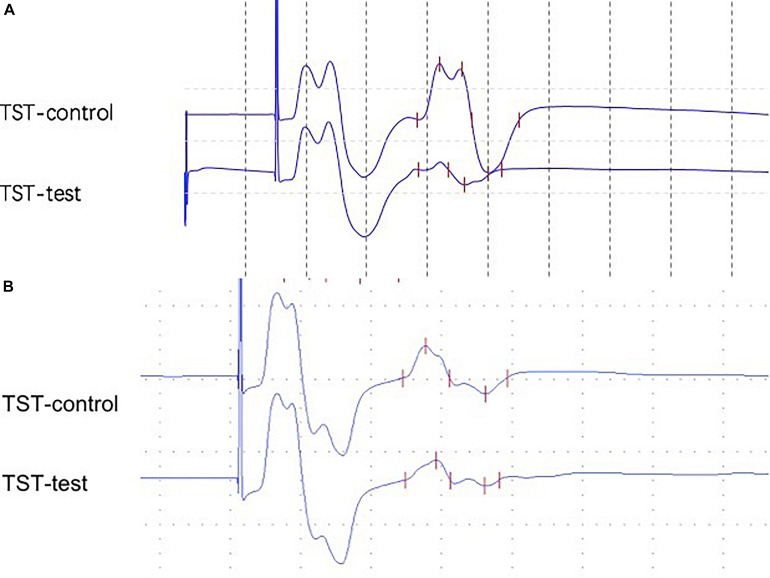
**(A)** Abnormal TST ratio (21.0%) from a 59-year-old female with a 2-year PD (patient 21). **(B)** Abnormal TST ratio (53.0%) from a 48-year-old male with a 1-year MSA-P (patient 2).

Triple stimulation technique has been well described in previous studies (Magistris et al., 1999; Groppa et al., 2012). Examples of the TST recordings are shown in Figure 1 (Magistris et al., 1998). Briefly, three stimuli with appropriate delays were given in a sequence as follows: TMS at the motor cortex, supramaximal electrical stimuli to the ulnar nerve at the wrist, and supramaximal electrical stimuli at Erb’s point. Two collisions occurred, and we obtained the TST test. The first delay was calculated as the MEP latency minus the compound muscle action potential (CMAP) latency (at the wrist). The second delay was Erb’s latency minus the CMAP latency (at the wrist). We then replaced the TMS at the motor cortex with the stimuli at Erb’s point with an adjusted delay and acquired the TST control curve. Finally, we calculated the baseline-to-negative peak amplitude ratio using the following formula: TST amplitude ratio = TST test amplitude/TST control amplitude.

### Statistical Analysis

The distribution of the categorical variables (gender, pyramidal signs, and H&Y stage) is presented as frequencies, and the continuous variables (age and disease duration) are presented as means ± standard deviations. A chi-square test was used for the categorical variables. For data with normal distributions, we used a one-way ANOVA and *post hoc* analyses to compare the TST ratios. All analyses were performed using the SPSS 22.0 software package.

## Results

### Subjects

The demographic and clinical characteristics of the subjects are summarized in Table 1. Age and gender were comparable between patients and HCs. The mean disease duration of MSA-P and PD was 2.71 and 2.90 years, respectively. There was no significant difference in disease duration or H&Y stage between MSA-P and PD patients. Patients were divided into three subgroups on the basis of the presence or absence of pyramidal signs: unilaterally present, bilaterally present, and bilaterally absent. There was no significant difference between MSA-P and PD patients in the presence of pyramidal signs (*p* = 0.393) or the percentage of pyramidal signs present (*p* = 0.581).

**TABLE 1:** Demographic and clinical characters of patients and healthy controls.

	**PD (*n* = 10)**	**MSA-P (*n* = 19)**	**HC (*n* = 12)**	***p*-value**
Age (years)	59.3 ± 4.4	57.6 ± 7.7	54.6 ± 9.6	0.347
Gender (male/female)	5/5	10/9	10/2	0.167*
Disease duration (years)	2.90 ± 2.69	2.71 ± 2.59	NA	0.855
Age of symptom onset	56.4 ± 3.7	55.1 ± 6.3	NA	0.549
Pyramidal sign (number)			NA	0.393*
Unilaterally present	1	6		
Bilaterally present	4	7		
Absent	5	6		
Percentage of pyramidal sign present^#^	45.0%	52.6%	NA	0.581*
H&Y stage			NA	0.067*
Stage 1	6	4		
Stage 2	4	11		
Stage 3	0	4		
Stage 4	0	0		
Average	1.4 ± 0.5	2.0 ± 0.7	NA	

These values represent the mean ± standard deviation or the number of patients. *Chi-squared test. #Percentage of pyramidal sign present = (number of unilaterally present + 2 × number of bilaterally present)/(the number of patient × 2) × 100%. PD, Parkinson’s disease; MSA-P, multiple-system atrophy parkinsonian variant; HCs, healthy controls; H&Y stage, Hoehn and Yahr stage; NA, not applicable.

### TST

Data obtained from TST are summarized in Table 2. All HCs presented with TST ratios within the normal range (≥90%). The TST ratio was significantly different among the three groups (*p* < 0.001). According to the *post hoc* analysis, PD patients had a significantly lower TST amplitude ratio (55.5 ± 32.2) compared with patients with MSA-P (*p* = 0.010) and HCs (*p* < 0.001). The TST ratio of patients with MSA-P (81.7 ± 19.8) was lower than that of HCs (96.8 ± 3.0), but this did not reach statistical significance (*p* = 0.160). Abnormal TST ratios (TST ratio <90%) were significantly more common in PD patients than in the other groups (*p* = 0.002). Representative TST curves of patients are shown in Figure 1. Detailed clinical and electrophysiological findings of patients and HCs are shown in Supplementary Table 1.

**TABLE 2:** Comparison of TST ratio in different groups.

	**PD (*n* = 10)**	**MSA-P (*n* = 19)**	**HC (*n* = 12)**	
TST ratio (%)	55.5 ± 32.2	81.7 ± 19.8	96.8 ± 3.0	
*P*-value*	0.000			
*P*-value (MSA-P and HC)	0.160			
*P*-value (PD and HC)	0.000			
*P*-value (MSA-P and PD)	0.010			
Percentage of abnormal TST ratio^#^	80.0%	36.8%	NA	*p* = 0.002

The TST ratio was presented as mean ± SD. *One-way ANOVA, *post hoc* analysis was performed using the Scheffe test. #TST ratio<90% was considered abnormal. Percentage of abnormal TST ratio = (number of unilaterally abnormal + 2 × number of bilaterally abnormal)/(the number of patient × 2) × 100%. MSA-P, multiple-system atrophy parkinsonian variant; PD, Parkinson’s disease; HCs, healthy controls; TST, triple-stimulation technique; NA, not applicable.

## Discussion

According to MDS-PD criteria, a parkinsonian patient with pyramidal signs might fulfill the diagnosis of clinically probable PD, provided that the patient has no absolute exclusion criteria or totals more than two red flags and has sufficient supportive criteria to counterbalance any red flags. All PD patients in this study were consistent with clinically probable PD. The presence of pyramidal signs in patients with PD and MSA-P in our study was 45 and 52.6%, respectively, which was not significantly different. Corticospinal tract impairment is common in patients with MSA-P. Pyramidal signs, which represent upper motor neuron (UMN) impairment, are considered one of the key clinical features of MSA-P (Eusebio et al., 2007). In a postmortem clinicopathological study of seven MSA-P cases, five patients had a positive Babinski sign. Furthermore, loss of Betz cells and presence of glial cytoplasmic inclusions in the primary motor cortex were confirmed by pathology examination in all cases, which was in accordance with UMN impairment (Tsuchiya et al., 2000). To the best of our knowledge, our study is the first to report pyramidal signs in PD.

The decreased TST ratios in our study also revealed profound corticospinal tract impairment in PD. Compared with MSA-P, TST ratio decreases in PD patients were much more prominent. A previous study reported cortico-spinal impairment in 50% of MSA-P patients, with a mean TST ratio of 86.6% (Eusebio et al., 2007). TST ratios were lower in MSA-P patients than HCs; however, this was not statistically significant. In our study, the average TST ratios of MSA-P patients and HCs were 81.7 and 96.8%, respectively, which is consistent with previous studies (Eusebio et al., 2007; Jang et al., 2014). Neuroimaging studies have demonstrated corticospinal tract impairment in patients with PD using DTI (Atkinson-Clement et al., 2017; Taylor et al., 2018). Increased FA in the motor tracts of PD patients suggests either compensatory neuroplasticity or selective neurodegeneration (Mole et al., 2016). To the best of our knowledge, this is the first study showing corticospinal tract impairment using TST.

Although TST ratio decreases in PD might be consistent with previous neuroimaging and electrophysiologic evidence, it was surprising that the TST ratio was much lower in PD patients than in MSA-P patients. Given that there was no significant difference in disease duration or H&Y stage between MSA-P and PD patients, this can only be explained by the updated diagnostic criteria for PD. Our TST ratio results were also lower than previous reports using PD patients as a control group. Jang et al. (2014) compared TST ratios of patients with PD, patients with vascular Parkinsonism (VP), and HCs. They found that the TST ratio of PD patients was similar to that of HCs (96.42 ± 5.11 vs. 97.70 ± 3.82%) and significantly higher than that of VP patients (71.59 ± 11.86%). Eusebio et al. (2007) reported that the TST ratio of PD patients was 99.1%, which was not different from HCs. Both of these studies were performed before the MDS-PD criteria were published; therefore, the pyramidal signs were absent. In contrast, pyramidal signs were present in almost half of the PD patients in our study, which might be directly associated with our TST findings.

There are shortcomings in our study. First, the sample size was limited. Further studies of TST with a larger sample are encouraged to explicitly investigate corticospinal pathway impairment in PD patients. Second, during the examination of pyramidal signs, no quantitative evaluation or interrater reliability analyses were performed, which may have introduced bias in the pyramidal sign results. Third, the neuroimaging methods used to determine abnormalities were performed in different hospitals. Therefore, the differences in scanning conditions may also have introduced bias. Repeated standardized neuroimaging data are necessary to fully exclude alternative causes of pyramidal signs in future studies. Finally, no neuroimaging sequence specific to corticospinal tract evaluation, such as DTI, was acquired.

In summary, our study showed profound decreases in TST ratios in patients with clinically probable PD. The results suggest that corticospinal tract impairment is not rare in PD and therefore warrants further observation and investigation. TST could be used to quantitatively evaluate corticospinal tract impairment and further our understanding of the pathophysiology of PD.

## Data Availability Statement

The raw data supporting the conclusions of this article will be made available by the authors, without undue reservation.

## Ethics Statement

The studies involving human participants were reviewed and approved by the Ethics Committee of Peking Union Medical College Hospital. The patients/participants provided their written informed consent to participate in this study. Written informed consent was obtained from the individual(s) for the publication of any potentially identifiable images or data included in this article.

## Author Contributions

DX: analyse the data and writing the article. QYD: perform all the TST and data collection. HW: design the study, recruit patients, and revise the article. All authors contributed to the article and approved the submitted version.

## Conflict of Interest

The authors declare that the research was conducted in the absence of any commercial or financial relationships that could be construed as a potential conflict of interest.
