# Supplementary material for: Corticospinal Tract Impairment of Patients With Parkinson’s Disease: Triple Stimulation Technique Findings
Source: Front Aging Neurosci. 2020 Nov 4;12:588085. doi: 10.3389/fnagi.2020.588085 (PMC7673408; doi:10.3389/fnagi.2020.588085)
Supplement: Supplementary file 1 [file Data_Sheet_1.docx]

**Supporting documents**

Here we present several examples of the patients which demonstrated the TST ration results.


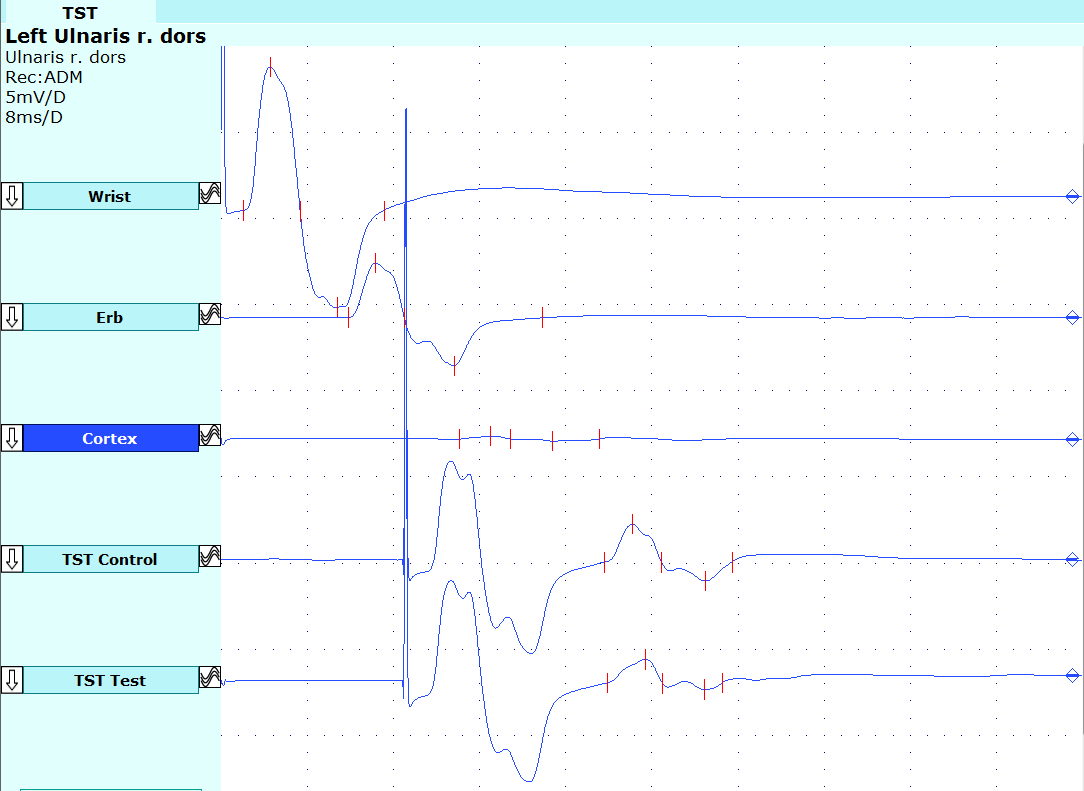


Fig 1. Original TST test and control curve of a 48-year-old male with a 1-year MSA-P (Patient 2, H-Y stage 2). TST ratio of the left side was 66.7%.


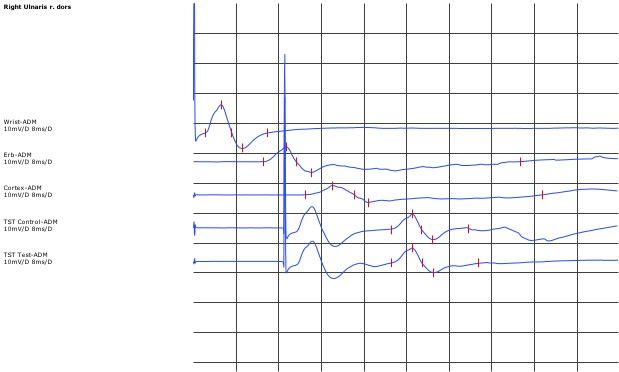


Fig 2. Original TST test and control curve of a 66-year-old male with 2-year MSA-P (Patient 4, H-Y stage 1). TST ratio of the right side was 98.8%.

**Left Ulnaris r. dors**

Wrist-ADM

5mV/D 8ms/D

Erb-ADM

5mV/D 8ms/D

Cortex-ADM

5mV/D 8ms/D

TST Control-ADM

5mV/D 8ms/D

TST Test-ADM

5mV/D 8ms/D

Fig 3. Original TST test and control curve of a 59-year-old female with 2-year PD (Patient 21, H-Y stage 1). TST ratio of the left side was 21.0%.
