# Supplementary material for: Corticospinal Tract Impairment of Patients With Parkinson’s Disease: Triple Stimulation Technique Findings
Source: Front Aging Neurosci. 2020 Nov 4;12:588085. doi: 10.3389/fnagi.2020.588085 (PMC7673408; doi:10.3389/fnagi.2020.588085)
Supplement: Supplementary file 2 [file Table_1.docx]

Table S1. Detailed clinical and electrophysiological findings of patients and healthy controls.

| Subjects’ number | Diagnosis | Gender^a^ | Age on TST | Age of onset | Duration（years） | Pyramidal sign(Right)^b^ | Pyramidal sign(Left)^b^ | H-Y stage | TST ratio(Right，%) | TST ratio(Left，%) |
| --- | --- | --- | --- | --- | --- | --- | --- | --- | --- | --- |
| 1 | MSA-P | M | 54 | 53 | 1 | - | + | 2 | 85.5 | 86.0 |
| 2 | MSA-P | M | 48 | 47 | 1 | - | + | 2 | 53.0 | 66.7 |
| 3 | MSA-P | F | 61 | 59 | 2 | + | + | 3 | 89.8 | 95.1 |
| 4 | MSA-P | M | 66 | 64 | 2 | - | - | 1 | 98.8 | 95.3 |
| 5 | MSA-P | M | 63 | 60 | 3 | - | - | 3 | 91.9 | 74.1 |
| 6 | MSA-P | M | 52 | 49 | 3 | + | + | 2 | 93.1 | 91.6 |
| 7 | MSA-P | M | 62 | 60 | 2 | - | - | 2 | 93.7 | 90.0 |
| 8 | MSA-P | F | 57 | 56 | 1 | - | + | 1 | 70.2 | 85.1 |
| 9 | MSA-P | F | 57 | 54 | 3 | + | + | 2 | 90.2 | 95.7 |
| 10 | MSA-P | M | 50 | 49 | 1 | - | - | 2 | 93.9 | 97.2 |
| 11 | MSA-P | M | 75 | 63 | 12 | + | + | 2 | 74.8 | 16.1 |
| 12 | MSA-P | F | 43 | 42 | 1 | - | - | 3 | 100 | 97.4 |
| 13 | MSA-P | F | 57 | 55 | 2 | + | - | 2 | 95.8 | 100 |
| 14 | MSA-P | F | 52 | 51.6 | 0.5 | - | - | 1 | 89.1 | 99.3 |
| 15 | MSA-P | F | 61 | 57 | 4 | + | + | 2 | 81.0 | 68.5 |
| 16 | MSA-P | F | 66 | 63 | 6 | - | + | 3 | 73.3 | 92.0 |
| 17 | MSA-P | F | 56 | 53.5 | 2.5 | + | + | 2 | 91.9 | 96.6 |
| 18 | MSA-P | M | 64 | 62 | 2 | + | + | 2 | 92.5 | 100 |
| 19 | MSA-P | M | 50 | 47.5 | 2.5 | + | - | 1 | 96.7 | 94.2 |
| 20 | PD | F | 58 | 56 | 2 | + | + | 1 | 70.4 | 45.9 |
| 21 | PD | F | 59 | 57 | 2 | - | - | 1 | 52.5 | 21.0 |
| 22 | PD | M | 57 | 54 | 3 | - | - | 2 | 97.0 | 92.1 |
| 23 | PD | M | 54 | 52.5 | 1.5 | + | + | 2 | 83.1 | 8.6 |
| 24 | PD | M | 52 | 51 | 1 | - | - | 1 | 82.5 | 86.4 |
| 25 | PD | F | 58 | 56 | 2 | - | - | 1 | 95.5 | 97.5 |
| 26 | PD | M | 62 | 61.5 | 0.5 | + | - | 1 | 88.6 | 78.8 |
| 27 | PD | F | 64 | 61 | 3 | + | + | 1 | 37.3 | 15.5 |
| 28 | PD | F | 64 | 54 | 10 | - | - | 2 | 80.6 | 56.2 |
| 29 | PD | M | 65 | 61 | 4 | + | + | 2 | 59.1 | 86.9 |
| 30 | HC | M | 54 | NA | NA | NA | NA | NA | NA | 100 |
| 31 | HC | M | 48 | NA | NA | NA | NA | NA | NA | 94.7 |
| 32 | HC | F | 61 | NA | NA | NA | NA | NA | NA | 100 |
| 33 | HC | M | 66 | NA | NA | NA | NA | NA | NA | 100 |
| 34 | HC | M | 63 | NA | NA | NA | NA | NA | NA | 94.4 |
| 35 | HC | M | 52 | NA | NA | NA | NA | NA | NA | 96.43 |
| 36 | HC | F | 62 | NA | NA | NA | NA | NA | NA | 94.7 |
| 37 | HC | M | 57 | NA | NA | NA | NA | NA | NA | 93.8 |
| 38 | HC | M | 57 | NA | NA | NA | NA | NA | NA | 92.6 |
| 39 | HC | M | 50 | NA | NA | NA | NA | NA | NA | 94.7 |
| 40 | HC | M | 75 | NA | NA | NA | NA | NA | NA | 100 |
| 41 | HC | M | 43 | NA | NA | NA | NA | NA | NA | 100 |

1. Gender: M=Male, F=Female
2. Pyramidal signs: “+” was defined as positive Babinski sign or Chaddock sign that documented in medical records, “-” was defined as negetive Babinski sign nor Chaddock sign that documented in medical records.
3. Early onset patients: Age of disease onset were before 50.

PD: Parkinson’s disease, MSA-P: multiple system atrophy parkinsonian variant, HCs: healthy controls, H-Y stage: Hoehn and Yahr stage. NA: Not Applicable. TST: triple stimulation technique.
